# Rhinolith: Delayed Presentation after Head Trauma—A Case Report

**DOI:** 10.1155/2012/492081

**Published:** 2012-12-13

**Authors:** Ali S. Al Mastour, Wagih M. Ghnnam, Abdu H. Zubaidi

**Affiliations:** ^1^Otolaryngology & Head and Neck Surgery Department, Khamis Mushayt General Hospital, Abha, Saudi Arabia; ^2^General Surgery Department, Mansoura Faculty of Medicine, Mansoura University, Mansoura, Egypt; ^3^General Surgery Department, King Khalid Hospital, Najran, Saudi Arabia

## Abstract

Rhinoliths are uncommon clinical entities reported in clinical practice as unusual cause of unilateral nasal obstruction and foul smell nasal discharge. Rhinolith is calcified material found in the nasal cavity incidentally or due to patient complaint. It should be suspected when patient presents with nasal symptoms and found to have stony mass showed radiologically. We reported a 28-year-old Saudi male with left sided (LT) nasal obstruction and foul smell discharge for 5 years suspected as being due to foreign body presence since childhood due to head trauma following car accident in sandy area.

## 1. Introduction

 Rhinoliths are calcified material around intranasal foreign body. They can be endogenous if occur around body tissues as tooth or exogenous if they occur around foreign subject as stones, cotton, or beads. They are found usually in anterior nasal cavity commonly associated with narrowing due to deviated septum, spurs, and/or turbinate hypertrophy. Endoscopic appearance is the main step in diagnosis which can be supported by radiology. Complete resolution of symptoms occurs after endoscopic surgical removal [1–3].

## 2. Case History

**Figure 1:**
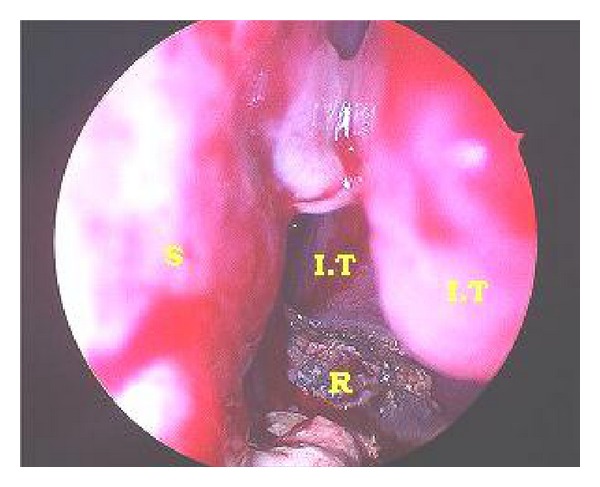
Rhinoscopy view of rhinolith (IT: inferior turbinate, S: septum, R: rhinolith).

**Figure 2:**
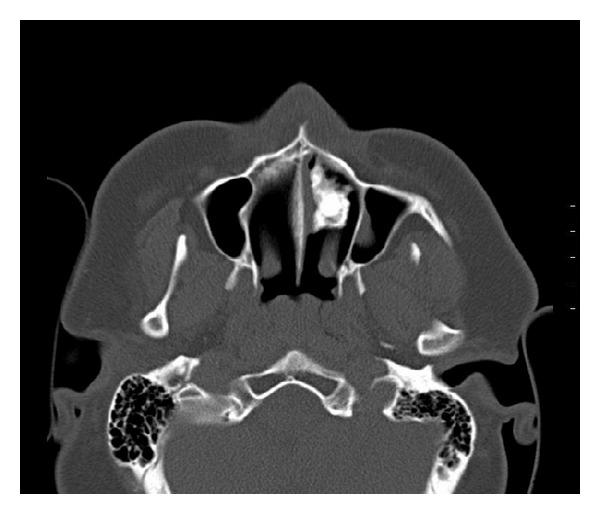
CT showing rhinolith.

**Figure 3:**
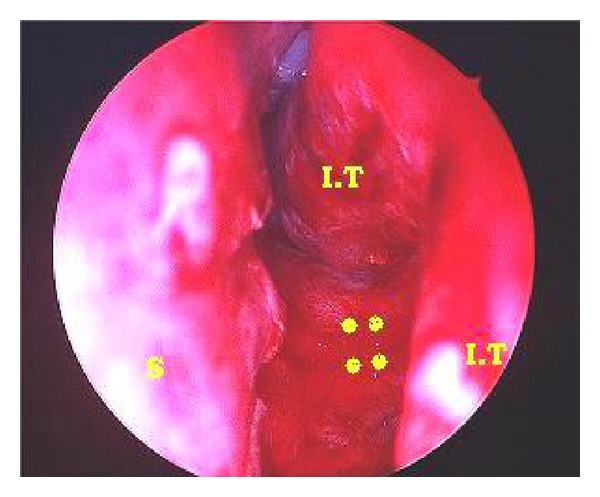
Rhinoscopy view after removal of rhinolith (IT: inferior turbinate, S: septum; yellow spots represent the site of rhinolith).

**Figure 4:**
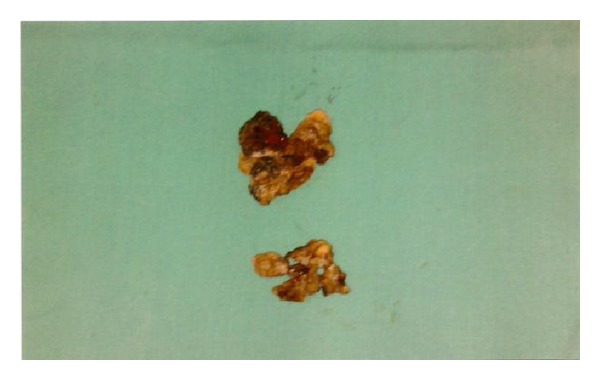
Rhinolith removed in two pieces.

A 28-year-old Saudi male presented to ORL HNS clinic referred from another hospital for his complaint of left (LT) nasal obstruction and foul smell discharge for five years. Symptoms were progressively noticed and disturb the patient's life in the last 3 years. He received multiple courses of antibiotics and nasal steroids with no benefit. He had no history of foreign body introduced into nasal cavity. He had history of head trauma after car accident at childhood in sandy area. Anterior rhinoscopy showed irregular hard material with crustations and thick secretions around, stuck between septal spur and inferior turbinate at LT anterior nasal cavity (Figure 1). Trials of removal in clinic failed causing epistaxis. Plain X-ray and CT scan showed dense irregular material at LT nasal cavity occupying floor without extension outside nasal cavity (Figure 2). Rhinolith was suspected then endoscopic removal done anteriorly after rhinolith was divided in two pieces and bleeding controlled (Figures 3 and 4). Then antibiotic ointment was applied in the place and patient given oral augmentin 625 mg three times daily along with nasal decongestant and analgesia for one week. Patient came to the clinic after one week later in better condition with dramatic improvement and resolution of symptoms.

## 3. Discussion

Bartholin first described rhinolith in 1654. Then more than 600 cases had been reported [4]. Pathogenesis of rhinolith is not clear; patient is mostly asymptomatic for years after foreign body introduced into nasal cavity, and as the size enlarges it causes nasal obstruction. Rhinolith needs time to be formed which is suggested to be around 15 years. Diagnosis is based on history and physical examination. Patients commonly present with nasal obstruction, rhinorrhoea, epistaxis, and sinusitis—other less common symptoms include facial pain and headache. As it gets bigger it compromises blood supply causing pressure necrosis then erosion and perforations of surrounded structures [1, 3–5].

Rhinolith was found frequently as incidental finding during rhinoscopy as irregular, hard dark mass with greenish foul smelling crustations around. Radiological investigations such as plain X-ray and CT scan can support diagnosis and direct the management. Maclntyre was the first to describe rhinolith radiographically in 1900 [6]. Rhinoliths may present with variable opacities depending on the nature of the origin. Differential diagnosis includes benign lesions as osteoma and odontogenic tumours and malignant lesions as osteosarcoma [2]. Medical Treatment wasnot shown to be effective in such problem. Treatment of choice is endoscopic surgical removal and in extremely rare conditions it needs external approach [4–6]. Usage of local antibiotics and occasionally systemic ones after removal could improve the recovery.

 To our knowledge this case is the first case reported here in Saudi Arabia and the most interesting is the long standing time it takes to present, and ultimately the final diagnosis was reached by clinical suspicion and endoscopic management is the standard modality of treatment.
